# The Avant Rehabilitation Program and Cerebrolysin for Treatment of Post-Stroke Dysphagia: A Case Report

**DOI:** 10.25122/jml-2019-1008

**Published:** 2019

**Authors:** Nguyen Minh Hien, Dang Phuc Duc

**Affiliations:** Stroke Department,Military Hospital 103, Hanoi, Vietnam

**Keywords:** dysphagia, stroke, rehabilitation

## Abstract

Dysphagia is recorded in approximately a third of ischemic stroke patients and is associated with malnutrition, aspiration, and pneumonia. We report a case study that assessed dysphagia (GUSS, Gugging Swallowing Screen), paralysis (MRC, Medical Research Council), and disability (mRS, modified Rankin Scale) in the AVANT program (Austrian Vietnamese Advancement Neurorehabilitation Treatment) - a collaboration program between Vietnam and Austria to standardize and systemize neurorehabilitation after stroke practice in Vietnam.

## Introduction

Dysphagia is recorded in 34.7% of ischemic stroke patients [1] and is associated with malnutrition, aspiration, and pneumonia [2]. Dysphagia was a significant independent variable predicting death and disability at day 90 [1]. Therefore, treatment of dysphagia plays a critical role.

**Figure 1:**
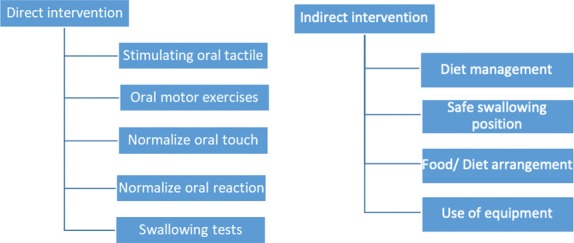
Dysphagia intervention through the AVANT program

The AVANT (Austrian Vietnamese Advancement Neurorehabilitation Treatment) program is a collaboration between Vietnam and Austria to standardize and systemize neurorehabilitation after stroke practice in Vietnam. The intervention procedure is divided into two groups: direct and indirect intervention (Figure 1).

## Case Report

**Figure 2:**
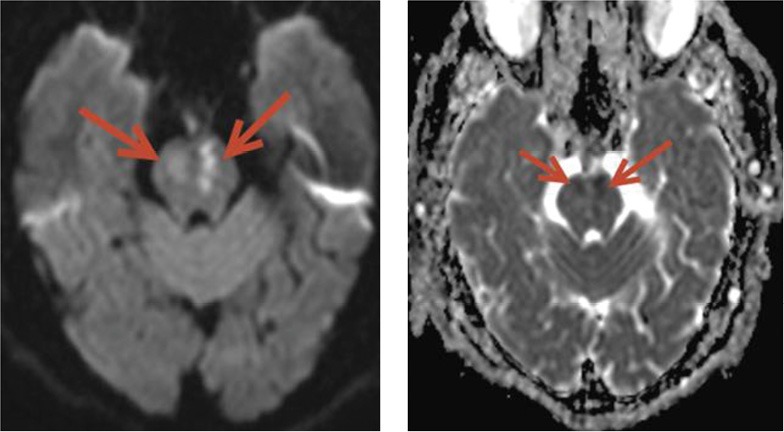
Brain MRI shows two areas affected by cerebral ischemia on the right and left part of the pons.

This case study refers to a 63-year-old male patient with a medical history of dyslipidemia and hypertension. In February 2019, the patient presented with left-sided hemiparesis, dysarthria, and dysphagia. He received antiplatelet drugs, diuretics and statins. There was a minor improvement in motor functions, but there was no improvement regarding swallowing. The patient was assessed as a high-risk patient for recurrent ischemic stroke with a low chance of recovery from dysphagia and a high chance of aspiration. Within three weeks, the patient suffered another ischemic stroke with right-sided hemiparesis, aggravation of dysarthria, and inability to swallow. A percutaneous feeding tube was inserted. The patient’s condition at hospitalization was described as:

– Conscious
– Dysphagia: Gugging Swallowing Screen (GUSS) score = 0; mandatory use of a feeding tube;
– Severe dysarthria: patient understood words but had difficulty in pronunciation; speech was difficult to understand;
– Tetraparesis: Medical Research Council (MRC) grade 2 for right and grade 3 for left extremities;
– No sphincter disturbance;
– Blood pressure: 120/75 mmHg, heart rate 75 bpm;
– modified Rankin Scale (mRS) score 4;
– Brain magnetic resonance imaging (MRI): two ischemic lesions in the left and right part of the pons (Figure 2).

The patient required intensive care and received the previously described treatment plus intravenous administration of 30 ml of Cerebrolysin per day.

Since the recovery of the swallowing function is challenging, yet vital to the patient, we decided to additionally involve a team of medical professionals trained in the AVANT program. This “recovery team” conducted a detailed evaluation of the patient’s dysphagia, designed an exercise program for muscles involved in swallowing (lips, sets of teeth, tongue, cheeks, fauces and so forth), assessed the condition of the patient daily, explained the patient and his family the mechanisms and causes of dysphagia, and gave them instructions on correct positions for eating and drinking, suitable types of food and drinks for each phase. In addition, rehabilitation therapy was initiated for the recovery of motor functions and communication. The patient was assessed for dysphagia (using GUSS), for motor deficits (using MRC) and disability (using mRS). 

*Day 7 (after the second stroke):* The feeding tube was removed on day 6. From day 7, the patient could eat semi-liquid food and sip 5 ml of water from a spoon. The medication was crushed and mixed with water to reach a slightly thinner consistency. The GUSS score was 11. The patient could pronounce 2-3 syllable words quite clearly. MRC grade 2 for right extremities, MRC grade 4 for left extremities, mRS 4.

*Day 14:* The patient could eat pieces of soft food and sipped 10 ml of water. The medication was crushed and mixed with water to reach a slightly thinner consistency. The GUSS score was 15. The patient could pronounce 2-3 syllable words quite clearly. MRC grade 3 for right extremities, MRC grade 5 for left extremities, mRS 4.

*Day 21:* The patient could slowly eat normal food. Medication in small tablets could be swallowed. The GUSS score was17. The patient could speak complete sentences, with difficulties, but the speech could be understood. MRC grade was 4 for right extremities and 5 for left extremities, with mRS 2. The patient could walk without assistance.

*Day 25:* The patient was discharged with mRS 2, and he was given instructions on the administration of drugs and exercises at home.

He received 30 ml of Cerebrolysin per day during the entire hospitalization period.

## Conclusion

In this case, the recovery of the swallowing function seemed difficult due to the severe dysphagia. However, the combined intervention of the AVANT program on swallowing function and the neurotrophic effects of Cerebrolysin as part of the treatment regimen resulted in improvements on dysphagia after the first week of treatment. By the end of the third week, considerable improvements were observed, and the patient was able to do daily activities such as walking, communicating, and eating without assistance. Early adoption of an appropriate rehabilitation program, combined with neurotrophic drugs such as Cerebrolysin, showed improved swallowing function in this stroke patient.

## Conflict of Interest

The authors confirm that there are no conflicts of interest.
